# Identification of production challenges and benefits using value chain mapping of egg food systems in Nairobi, Kenya

**DOI:** 10.1016/j.agsy.2017.10.001

**Published:** 2018-01

**Authors:** Joshua Orungo Onono, Pablo Alarcon, Maurice Karani, Patrick Muinde, James Miser Akoko, Carron Maud, Eric M. Fevre, Barbara Häsler, Jonathan Rushton

**Affiliations:** a Department of Public Health, Pharmacology and Toxicology, University of Nairobi, Kenya; b Leverhulme Centre for Integrative Research in Agriculture and Health, London, United Kingdom; c Department of Pathobiology and Population Sciences, Veterinary Epidemiology, Economics and Public Health group, Royal Veterinary College, University of London, United Kingdom; d International Livestock Research Institute, Kenya; e University of Liverpool, United Kingdom

**Keywords:** Layer production, Indigenous chicken, Urban farming, Public health risks, Value chain mapping, Nairobi

## Abstract

Commercial layer and indigenous chicken farming in Nairobi and associated activities in the egg value chains are a source of livelihood for urban families. A value chain mapping framework was used to describe types of inputs and outputs from chicken farms, challenges faced by producers and their disease control strategies. Commercial layer farms were defined as farms keeping exotic breeds of chicken, whereas indigenous chicken farms kept different cross breeds of indigenous chicken. Four focus group discussions were held with producers of these chickens in peri-urban area: Dagoretti, and one informal settlement: Kibera. Qualitative data were collected on interactions between farmers, sources of farm inputs and buyers of poultry products, simple ranking of production challenges, farmers' perception on diseases affecting chicken and strategies for management of sick chicken and waste products. Value chain profiles were drawn showing sources of inputs and channels for distribution of chicken products. Production challenges and chicken disease management strategies were presented as qualitative summaries. Commercial layer farms in Dagoretti kept an average of 250 chickens (range 50–500); while flock sizes in Kibera were 12 chickens (range 5–20). Farms keeping indigenous chicken had an average of 23 chickens (range 8–40) in Dagoretti, and 10 chickens (range 5–16) in Kibera. Commercial layer farms in Dagoretti obtained chicks from distributors of commercial hatcheries, but farms in Kibera obtained chicks from hawkers who in turn sourced them from distributors of commercial hatcheries. Indigenous chicken farms from Dagoretti relied on natural hatching of fertilised eggs, but indigenous chicken farms in Kibera obtained chicks from their social connection with communities living in rural areas. Outlets for eggs from commercial layer farms included local shops, brokers, restaurants and hawkers, while eggs from indigenous chicken farms were sold to neighbours and restaurants. Sieved chicken manure from Dagoretti area was fed to dairy cattle; whereas non-sieved manure was used as fertilizer on crops. Production challenges included poor feed quality, lack of space for expansion, insecurity, occurrence of diseases and lack of sources of information on chicken management. In Kibera, sick and dead chickens were slaughtered and consumed by households; this practice was not reported in Dagoretti. The chicken layer systems contribute to food security of urban households, yet they have vulnerabilities and deficiencies with regard to disease management and food safety that need to be addressed with support on research and extension.

## Highlights

• Commercial layer farms obtained chicks from hatcheries and from hawkers
• Indigenous chicken farms relied on natural hatching of fertilised eggs
• Sieved chicken manure fed to dairy cattle; whereas non-sieved manure is used as fertilizer on crops
• Challenges include poor feed quality, lack of space for expansion, insecurity and diseases
• Sick and dead birds were slaughtered and consumed by households in informal settlement

## 1 Introduction

Poultry keeping is an important livestock enterprise practised by most Kenyan households (Behnke and Muthami, 2011). In 2014, the contribution of poultry offtake and egg production to the national agricultural gross domestic product was estimated at 1.3% (USD 46.16 million) and 2.9% (USD 103.05 million), respectively (KNBS, 2015). In 2009, the national poultry population was estimated to be 32 million birds (Behnke and Muthami, 2011) with the majority (84%) being free-ranging indigenous chicken, with smaller numbers of commercial layers (8%), commercial broilers (6%) and other species such as ducks, turkeys, pigeons, ostriches, guinea fowls and quails (2%) (Behnke and Muthami, 2011, FAO, 2007). It was reported that every rural Kenyan household keeps indigenous chickens with an average flock size of 12 (CTA, 2007, Kingori et al., 2010). The major concentration of commercial layers was found in Nairobi County (180,000 birds), in addition to an estimated 260,000 indigenous chickens kept in this County (GoK, 2012). Indigenous chicken kept in Kenya have been described using phenotypic characteristics: fizzled feathered, naked neck, barred feathered, feathered shanks, bearded and dwarfed size (Kingori et al., 2010). These indigenous chickens are a result of uncontrolled cross breeding programmes between various lines of local and exotic breeds of chicken.

Egg production from commercial layers and indigenous chicken is important both in terms of meeting nutritional needs of Kenya, as well as an economic activity. Chickens are a reliable source of nutritious food and income to many resource poor households and are relatively easy to rear. Chicken eggs have high levels of micronutrients including carbohydrates, fats and fatty acids, protein and amino acids, vitamins (D and B12) and minerals (Exler et al., n.d.). According to the report on recommended dietary allowances of the Food and Nutrition Board of the United States, eggs are considered to be rich in essential amino acids: histidine, isoleucine, leucine, methionine and cysteine, lysine, phenylalanine and tyrosine, threonine, tryptophan, and valine (National Research Council, 1989). Based on this report, eggs contain 490 mg/g of the essential amino acids which is above the dietary requirements for infants aged 3 to 4 months old (412 mg/g), 2 years old (320 mg/g), 10–12 years old (220 mg/g), and adults (111 mg/g). Furthermore, the report states that digestibility of egg protein in human is approximately 100%. Other studies have further reported that chicken eggs contain approximately the same amount of animal protein as pork, poultry meat, beef and whole milk cheese (Ondwasy et al., 2006, Seidler and Martin, 2003). Therefore, in urban communities with limited access to land, chicken rearing represents an alternative source of high quality nutrition for poor households.

These positive aspects need to be balanced against possible problems with disease management, particularly of diseases that affect both poultry and humans. Some infectious diseases which affect poultry in Kenyan farms include salmonellosis and Newcastle disease, while risk factors for occurrence of avian influenza have been described (FAO, 2007, Kingori et al., 2010, Nyaga, 2007). Furthermore, organisms like *Salmonella pullorum* and *Salmonella gallinarum* can colonise the reproductive system of chickens and can be transmitted through eggs to chicks which are replacement stock (Ribeiro et al., 2009). Apart from their effect on lowered farm productivity, poultry diseases present potential public health risks to consumers of poultry products and people in contact with infected farms (Duffy et al., 2012, Kariuki et al., 1997, Lee and Newell, 2006, Svetoch and Stern, 2010).

It also needs to be recognised that the Kenyan poultry sector is changing with greater demands for livestock products in urban areas that are both growing in size and wealth. This indicates that both the positive aspects of the poultry sector (i.e. nutrition and income) and the potential negative externalities (i.e. public health risks) are changing. However, there is a paucity of information on productivity and profitability of the commercial layer and indigenous production systems and their associated value chains in Kenya in general and in urban settings in particular. There is also a lack of information on disease risks generated by these systems and how they are managed within the production systems and value chains. Therefore, the aim of the study was to map value chains for eggs from commercial layer and indigenous chicken farms and identify practices which increase public health risks within the egg supply chains in Nairobi. The results will assist policy makers in understanding challenges that commercial layer and indigenous chicken farmers face within *peri* urban areas and informal settlements.

## 2 Materials and methods

### 2.1 Research design and study area

**Fig. 1:**
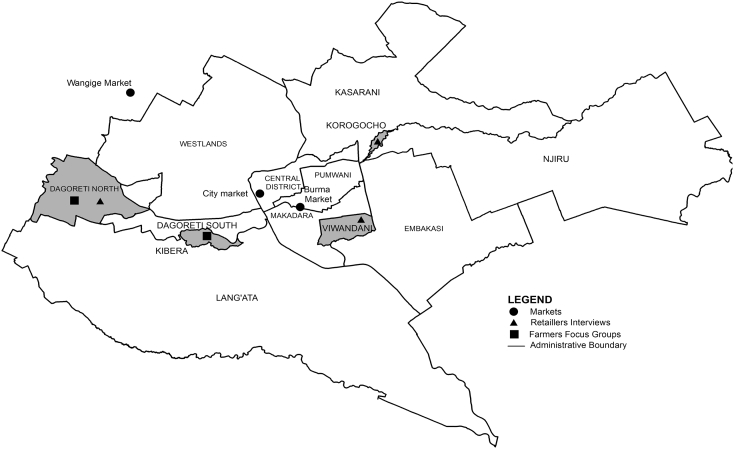
Map of Nairobi County showing the selected study areas.

A descriptive study was conducted in 2014 in two areas within Nairobi (Fig. 1). The informal settlement: Kibera, located in Langata sub-County and the peri-urban area: Dagoretti, located in Dagoretti North sub-County. Inhabitants of Kibera migrated from other parts of the country to seek better jobs in the city, and have brought with them livestock farming practices, while in Dagoretti, the natives have practised livestock farming for ages. These areas were purposively selected for different reasons: According to government livestock officers Dagoretti has the largest number of livestock farming activities in Nairobi. Kibera, which is the largest informal settlement in Nairobi, was selected because of the co-existence of commercial layer and indigenous chicken farms. The choice of descriptive study design was considered useful for expanding the understanding and insight in commercial layer and indigenous chicken farming practices in Nairobi, which could support formulation of hypotheses for future studies under similar production systems (Kothari and Garg, 2014). The framework adopted for mapping of chicken system was used in a previous study which mapped systems structures and flows for beef, sheep and goats to identify deficiencies and vulnerabilities to food system shocks in Nairobi (Alarcon et al., 2017).

### 2.2 Selection of study participants and data collection

A list of chicken farmers within the study areas was not available. Therefore, local livestock extension and veterinary officers were asked to identify and recruit commercial layer and indigenous chicken farmers for the study. The livestock extension officers were requested to recruit representatives of chicken farmers based on the scale of production: small scale, medium scale and large scale. The selected farmers were invited to attend focus group discussions which were held in Dagoretti and Kibera areas. In each area, separate discussions were held with farmers of commercial layer and indigenous chicken. The group discussions were conducted in Kiswahili language which all participants understood. These discussions were led by a member of the research team who wrote the main points on a flip chart so that all participants could discuss and reach consensus on different responses which were provided. Data were collected on: (1) flock sizes and farmers' perceptions on types of production systems practised (either layer or indigenous chicken farms), (2) types of farm inputs (these included sources of day old chicks, mature birds, water, types and sources of feed and veterinary supplies and services), (3) type of farm outputs (these included products derived from farms, their buyers and farm gate prices associated with each buyer), (4) chain governance (participants were asked why they used different input sources, output outlets and services, membership of associations, interaction with government officers in animal health management, buying and selling of day old chicks, future aspirations of chicken farmers and, management of waste products), (5) chicken production challenges, (6) chicken farmers' perception on diseases affecting their flocks, and strategies employed when handling sick and dead birds, and the risk points for poultry diseases and food safety. In addition, participants were asked to rank the chicken production challenges identified using a numerical scale and to reach a group consensus using simple ranking method. Different retailers were visited to triangulate the data on buying prices for eggs and spent laying hens. Livestock production officers from both Dagoretti North and Langata sub-Counties were interviewed to validate findings from chicken farmers on the types of production challenges and poultry diseases that farmers face. Ethical clearance certificates for this research was issued by the ethics and welfare committee of the Royal Veterinary College, University of London (Reference number URN 2013 0084H); and International Livestock Research Institute (ILRI) Institutional Research Ethics Committee (Reference number ILRI-IREC2014-04).

### 2.3 Data management and analysis

Data that were collected from focus group discussions and key informant interviews were recorded on flip charts, note books and in audio recordings. These data were transferred to document templates prepared in Microsoft Word. These templates constituted an initial step in analysis where data were structured and entered in sections corresponding to each question which was investigated based on the framework analytical approach (Gale et al., 2013). The framework approach is a systematic search for patterns to gain full descriptions necessary for shedding light on a topic under investigation. Several salient themes were identified at this stage by carefully listening to audio recordings, and these were entered in templates. Thematic analysis was done to identify patterns relating to themes which influenced the use of different sources of farm inputs, buyers of chicken products and sources of information on chicken management. Value chain profiles (flowchart diagrams) were drawn based on the information obtained on sources of chicken and farm inputs (water and feed), and the destination of products as reported and agreed by farmers in their focus groups. The profiles were created by identifying flow of inputs into chicken farms, types of farms classified by scale of production and number of birds kept in each area and outflow of products from each production system. This approach has been described in a related publication which mapped beef, sheep and goat systems in Nairobi (Alarcon et al., 2017). Discount rates offered to traders who purchased products at farm gate was calculated as the difference between the actual price paid by traders for a product and market price expressed as percentage of the market price (Exchange rate 1 USD = KSh 100). Management practices and strategies which were applied by chicken farmers in handling of sick and dead birds and waste disposal on farms, their knowledge of diseases affecting flocks, and challenges that affected chicken production and their corresponding ranks were presented as narrative summaries based on the different themes.

## 3 Results

### 3.1 Mapping of value chains for chicken products

**Fig. 2:**
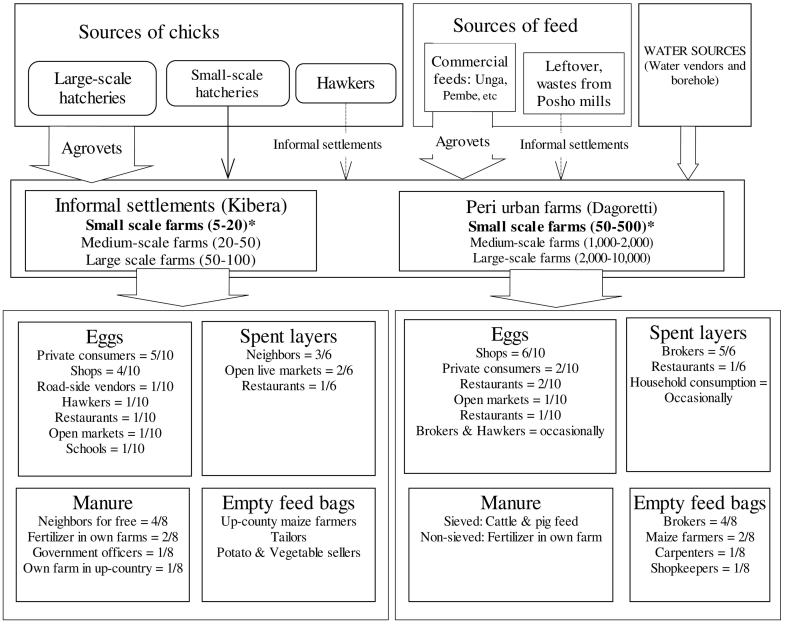
Value chain map for input and products from commercial layer farms in Nairobi.

**Fig. 3:**
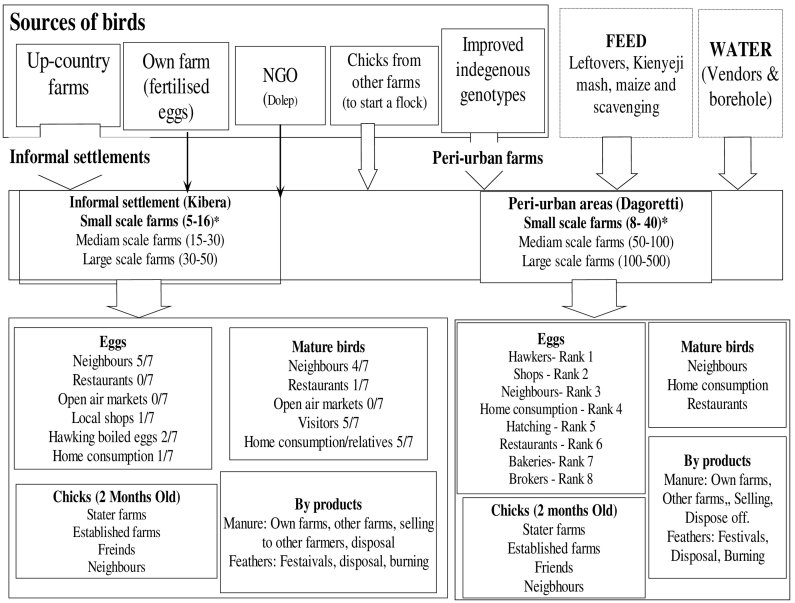
Value chain map for inputs and products from indigenous chicken farms in Nairobi.

A total of 38 chicken farmers participated in the focus group discussions with an average of 9 chicken farmers participating in each group discussion (Table 1). Two value chain profiles were drawn, one for commercial layers and another for indigenous chickens as shown in Fig. 2, Fig. 3. There were differences in the number of chickens kept per farm in the two study areas. In farms keeping commercial layers, an average of 250 chickens (range 50–500) per farm were kept in Dagoretti, and 12 chickens (range 5–20) per farm within Kibera. Indigenous chicken farmers kept an average of 23 chickens (range 8–40) per farm in Dagoretti and 10 chickens (range 5–16) per farm within Kibera. Although participants reported that large scale farms in the areas would keep between 2000 and 10,000 chickens, none of the participants owned this flock sizes.

**Table 1:** Participants of focus group discussions and key informant interviews held in the study area of Kibera and Dagoretti.

Area of study	Focus group discussions	Key informant interviews
Kibera	Commercial layer farms − 6 females and 4 males Indigenous chicken farms − 2 females and 5 males	2 males (Livestock production officers)
Dagoretti	Commercial layer farms − 6 females and 3 males Indigenous chicken farms − 6 females and 4 males	2 female and 1 males (Livestock production officers)

Sources of day old chicks for commercial layer farms included commercial hatcheries whose day old chicks were sold through distributors, i.e. shops selling agricultural inputs. However, farms from Kibera obtained day old chicks from hawkers (retailers who move with their merchandise between markets who in turn sourced chicks from distributors of commercial hatcheries). Farmers who kept indigenous chicken within informal settlements in Kibera obtained chicks from their social connections with communities living in rural areas. Yet, farmers of indigenous chicken from Dagoretti relied on other sources including neighbouring farms and non-governmental organisations which support rearing of indigenous chicken.

Most commercial layer birds kept in Dagoretti were fed on commercial feed purchased from shops selling agricultural inputs. However, those kept in Kibera and indigenous chicken from both areas were fed on leftovers and posho mill wastes. In both areas, most of the indigenous chickens were scavenging for food. Besides, these birds were frequently supplemented with leftovers (wastes from vegetable markets, kitchen wastes) and occasionally with commercial feeds. Farms under both production systems mostly watered birds using water from boreholes and water vendors.

Chicken products were sold to various outlets: local shops, brokers, neighbours, hawkers, food restaurants, and open air markets (Fig. 2, Fig. 3). The main products sold were eggs and spent laying hens. Indigenous chicken farms also had other products: Cockerels and 2 month old chicks for sale to other farmers. Results showed that majority of commercial layer farms from Dagoretti sold eggs to local shops, while those from Kibera sold eggs to their neighbours for private consumption and local shops. Indigenous chicken farmers from Dagoretti sold eggs to hawkers, but in Kibera, these were sold to neighbours. Other outlets for eggs from indigenous chicken included hawkers, local shops, bakeries and private consumers. Spent laying hens from commercial layer farms from Dagoretti were sold to brokers, but in Kibera these were sold to neighbours and in local open air markets. Farmers of indigenous chicken in both areas always sold mature birds to neighbours, gave them as gifts to visitors and used them for home consumption. Only one participant reported to sell their eggs to a restaurant. By-products from these systems included manure, feathers and empty feed bags. Manure was dried and sieved for use as feed for dairy cattle, and used when not sieved as crop fertilizer. Empty feed bags were purchased by traders: brokers, furniture workshops, maize farmers and retail shops. Chicken feathers were disposed off in surroundings of homesteads, but farmers of indigenous chicken often used them for preparing traditional dancing costumes for school children. Furthermore, some individuals occasionally purchased quality feathers from the back of indigenous chicken for making fishing hooks.

### 3.2 Factors influencing choice of suppliers, sellers and sources of information

Factors which were mentioned by commercial layer farmers as important when selecting their sources of chicks included: hatcheries whose chicks have a history of low rates of mortality and morbidity, birds that start laying eggs at the age of 4.5 months, birds that produce bigger sized eggs, birds with small stature which consume less feed, low purchase price for day old chicks, birds laying eggs for longer periods, proximity to a large company's distribution point and the reliability of large companies in supply of day old chicks. For indigenous chicken, no reason was obtained as most supply was from their social connections with communities living in the rural areas.

Restaurant owners often gave verbal contracts to commercial layer farms to supply eggs, but neighbours who bought eggs for household consumption preferred to buy directly from farms. Food restaurants had preference for yellow yolk normal sized eggs from indigenous chicken farms, while small sized eggs were sold to brokers who often mixed them with bigger eggs to increase their gross margins. Eggs with weaker shells and those which were cracked were sold to neighbours and hawkers who would use them for home consumption, or would boil them and sell by the roadside. Also, eggs that remained after selling to other traders were sold to hawkers. Peak season for eggs sales was in the dry season when prices for green vegetables were high and eggs were used as substitute product. During peak season, the price per tray of 30 eggs was KSh 300 or more, but during low season (rainy season) prices could be less than KSh 200 per tray. The farmers had no access to formal training on chicken management, but they occasionally obtained information from fliers and pamphlets from large companies operating hatcheries. Other sources of information included farmers and company agents who provided information on chicken feeding, diseases and their control. Government extension officers were rarely consulted because they were perceived to provide unreliable services. Farmers of indigenous chicken reported to have limited or no interaction with government extension officers. Moreover, commercial layer and indigenous chicken farmers did not belong to organised associations. According to these farmers a few large companies had dominated sale of chicks, while egg traders at a local market “Wangige” had influence on setting market prices for eggs.

Discount rates offered to traders who purchased products at the farm gate for commercial layer and indigenous chicken farms respectively, are shown in Table 2, Table 3. Owners of restaurants, brokers and retailers at open air markets obtained higher discount rates per tray of 30 eggs from commercial layer farms as compared to neighbours, hawkers and owners of local shops. Similarly, brokers and owners of restaurants obtained high discount rates on spent laying hens.

**Table 2:** Farm gate prices and discount rates offered on products from indigenous chicken farms in Nairobi.

Traders	Tray of 30 eggs (KSh)	KSh per spent layer	2 months old chick (KSh)	Cockerel (KSh)
Market prices	600	1000	300	1500
Neighbour	450 (25%)	600 (40%)	250 (20%)	700 (53%)
Shop	450 (25%)	–	–	–
Bakery	450 (25%)	–	–	–
Hawker	450 (25%)	–	–	–
Broker	300 (50%)	–	–	–
Restaurant	600 (0%)	500 (50%)	–	600 (60%)
Visitor	600 (0%)	600 (40%)	–	700 (53%)
Friend	–	–	250 (20%)	–
Relative	–	500 (50%)	–	500 (67%)

Exchange rate: 1 USD = 100 KSh.

**Table 3:** Farm gate prices and discount rates offered on products from commercial laying farms in Nairobi.

Traders	Tray of 30 eggs (KSh)	Spend laying hens (KSh)
Market prices	300	450
Restaurant	260 (13%)	400 (11%)
Local shop	275 (8%)	300 (33%)
Brokers	260 (13%)	200 (56%)
Neighbour	300 (0%)	350 (22%)
Market	260 (13%)	350 (22%)
Hawker	300 (0%)	–

Exchange rate: 1 USD = 100 KSh.

### 3.3 Description and ranking of challenges for layer production

Challenges which were identified by farmers of commercial layers and indigenous chicken are shown in Table 4, Table 5, respectively. Commercial layer farmers within Kibera ranked ‘lack of land/space for keeping birds’ first, while this was not reported as a challenge by farmers in Dagoretti. Within Kibera, layer farmers ranked incidence of insecurity second, which was only ranked eighth in Dagoretti. The main challenge for commercial layer rearing within Dagoretti was related to lack of training. For indigenous chicken farms, lack of land/space was ranked first within Kibera and third in Dagoretti. Lack of passion for keeping indigenous chicken by younger members of the community was a major challenge identified in Dagoretti. Other challenges identified to affect chicken farming within the studied systems included unavailability of chicks, poor feed quality, lack of training centres and sources of information for chicken rearing, shortages of labour, poor water quality, high capital outlay, high costs of feeding and water troughs, insecure chicken housing, and high costs of veterinary drugs. Unavailability of feed was identified as a challenge, partly because of value added tax which was charged by national government on agricultural inputs. Only a few companies were selling quality feeds, but others had mushroomed which manufactured poor quality feeds. Feed of poor quality was blamed for reduced egg production and slower growth rates in chicken. Participants reported increased frequency of disease occurrences during cold seasons. Other causes of disease which were reported included poor hygiene in farms and allowing visitors in chicken housing. Farmers had different aspirations for their businesses. For example, indigenous chicken farmers in Dagoretti listed increasing flock sizes; commercialization of production; and constructing bigger poultry housing, while those from Kibera listed becoming large-scale farmers, sourcing for good cockerels and capital for expansion of business. For the commercial layer farms, those from Dagoretti listed increasing flock sizes; buying of vehicles to support their business activities, manufacturing own feed, owning hatcheries, sourcing for capital to support expansion; and forming associations, while those from Kibera listed becoming large-scale farmers; obtaining knowledge on chicken management; and acquisition of incubators.

**Table 4:** Production challenges ranked by farmers of indigenous chicken in Dagoretti and Kibera in Nairobi.

Challenge	Rank		Implications for indigenous chicken farming in Nairobi
	Dagoretti	Kibera	
Lack of motivation	1	–	Lack of motivation on keeping indigenous chicken by young people
Insecurity in farms	2	2	Theft of chicken linked to types of housing and malicious behaviour towards chicken
Limited land/space	3	1	Indigenous chicken kept on free range and dense human population exposing them to disease
Sources of Chicks	4	4	Farmers hatching own fertilised eggs or buying already hatched eggs/chicks from other farms
Poor quality feeds	5	3	Poor quality feeds e.g. “Kienyeji” chicken mash, market leftover from informal settlements, kitchen leftovers, posho mill wastes and vegetable wastes from markets fed to chicken
Lack of information/training	6	8	Limited training only offered to farmer groups and not individual chicken farmers
High cost of equipment's e.g. feeding troughs etc.	–	5	–
Water shortage	–	6	Birds watered from water sourced from vendors and boreholes
Shortage of labour	–	7	Labour was often obtained from family members and hired employees from outside Nairobi
Large capital needs	–	–	Capital was mainly from personal savings

**Table 5:** Production of challenges ranked by farmers of commercial layers in Dagoretti and Kibera in Nairobi.

Challenge	Rank		Implications for commercial layer farming in Nairobi
	Dagoretti	Kibera	
Lack of land/space	–	1	Birds producing bad smell and noise which affects neighbouring households
Insecurity	8	2	High cases of theft of chicken
Sources of information	1	3	Information on poultry rearing only obtained from other farms and company agents who give brochures containing information on poultry feeding and disease control
Sources of feeds	2	5	Unlicensed feed manufacturers mushrooming within the country, very high feed prices; poor quality feeds leading to low performance i.e. fewer eggs and slows growth rates
Lack of capital	3	–	–
Types of housing	4	–	–
Sources of chicks	5	–	
Cost of veterinary drugs	6	–	–
Cost of energy e.g. electricity/charcoal	7	6	–
Cost of equipment: e.g. sawdust, feeders, drinker		7	–

### 3.4 Farmer's knowledge of chicken diseases and their response to outbreaks

Diseases that frequently affected flocks in the study areas according to the farmers included diarrhoea, coccidiosis, infectious bursal disease, flea and worm infestation, Newcastle disease, calcium deficiency, cannibalism, swollen eyelids and necks and respiratory problems. According to these farmers, veterinary medicines were obtained from shops selling agricultural inputs, while government veterinary services were rarely used. About half of commercial layer and most indigenous chicken farmers from Kibera reported slaughter of sick birds, and they would either sell or consume the meat. Conversely, commercial layer and indigenous chicken farmers from Dagoretti would take sick or dead birds to nearest shops selling agricultural inputs to seek advice on how to treat the remaining sick birds. Other strategies used by farmers for handling sick and dead birds are listed in Table 6. Disease prevention measures practiced in commercial layer farms in Dagoretti included vaccination, construction of footbaths for personnel in poultry housings, keeping visitors away from chicken housings, heating of chicken houses during cold seasons and having protective clothing and shoes designated for visiting chicken houses. However, some practices by farmers of commercial layer and indigenous chicken constituted food safety risks. These included sale of chicken and eggs without observing withdrawal period for antibiotics, sale and consumption of sick and dead birds, handling of chicken manure without protective clothing, slaughtering birds in non-designated places, hence leaving blood and other slaughter wastes within environment of homesteads.

**Table 6:** Strategies practised by chicken farmers when handling sick and dead birds in Nairobi.

System/farms	Peri-urban areas (Dagoretti)	Informal settlements (Kibera)
Commercial layer	Seek advice from veterinarians and hatcheries; isolation of sick birds; slaughter sick birds before they die; take sick birds to “agrovets” and seek advice on treatment of remaining birds in flock, dead birds are boiled and fed to dogs; selling of sick birds at reduced price	Burying dead birds; given to garbage collectors; home consumption; slaughter before sick birds die; disposal in dumping sites; when they are many “slaughter and roast as a form of preservation”; in case birds have watery and foul smelling diarrhoea they are treated with *Aloe vera* plant extracts (medicinal plant)
Indigenous chicken	Burying dead birds within homesteads; taken to “agrovets” for advice on how to treat birds remaining in flocks	Slaughter sick bird before dies; home consumption; burning of dead birds

## 4 Discussion

This report has presented value chain profiles showing the flow of inputs to chicken farms and products to market outlets. The profiles show relationships between people who were connected through activities associated with chicken production, including suppliers of farm inputs and customers of chicken products. These networks provide a framework through which benefits of chicken production and vulnerabilities associated with chicken enterprises can be examined. Furthermore, understanding these networks is important for policy makers to consider measures for disease control.

The study demonstrated that egg laying birds play a significant role in the provision of eggs to households in peri-urban and informal settlements in Nairobi. Apart from eggs, these laying birds also provided a source of income from sale of spent hens, chicks sold to starter farms and chicken manure which was used either as fertilizers on crop farms or as feed supplement for dairy cattle. These are important roles that chicken plays in supporting livelihoods of vulnerable households. In a similar study which was conducted in Uganda, it was reported that vulnerable households living in rural areas depended on proceeds from sale of chicken to support their healthcare, payment of school fees and purchase of other household necessities (FAO, 2009).

Key inputs to chicken systems included water, feeds, veterinary services and replacement birds. The mechanisms for supply of farm inputs present biosecurity concerns to connected farms and systems. For example, transportation of indigenous chicken from rural villages to farms within informal settlement is a potential source for introduction and spread of infectious diseases. This proposition is supported by farms of indigenous chicken from informal settlement obtaining replacement stock from their social connections with communities living in rural areas. This is further aggravated by practices of feeding leftovers to commercial layers and scavenging of indigenous chicken within informal settlement, where interaction between people and chicken was high due to dense population and land tenure problems. Indigenous chicken were kept for household consumption, although some products were sold to neighbours. Their role was restricted to household food security, besides cultural functions associated with by-products like feathers. Therefore, this system has limited prospects for improvements especially within informal settlement. Furthermore, scavenging by these chickens would expose them to contaminated feed and water sources, which compromises biosecurity between connected farms (Conan et al., 2012, Margaret et al., 2015, Sultana et al., 2012, Van Kerkhove et al., 2009). Indeed, outbreaks of diseases like salmonellosis, Newcastle disease and highly pathogenic avian influenza (*HPAI*) have been associated with uncontrolled movement of birds between connected farms and systems (Chotinun et al., 2014, Conan et al., 2012).

Chicken products were also marketed through different market outlets, which had significant influences on producers. In the present study, several people had important influences on egg producers. For example, supply of day old chicks was controlled by few companies, based on their superior breeds, which were distributed through shops selling agricultural inputs, making them instrumental in the process, while local shops were the main buyers of quality eggs from these farms. Conversely, the practice of selling cracked eggs and those with weaker shell to hawkers could be a source of food safety risk to consumers of boiled eggs. Therefore, implementation of effective policies and strategies for control of infectious diseases in peri-urban and informal settlement areas should consider these influences on egg production.

The strategies adopted by farmers to manage disease occurrence in chicken or handling of dead birds may result in spread of diseases between affected systems and connected farms. For example, the practices of selling and consumption of sick birds, and disposal of dead birds in open sewerage systems and damping sites are potential health risks. These are important environmental contamination, because zoonotic pathogens can persist in the environment. For example, *Salmonella* species was demonstrated to persist longer in untreated water which was collected from poultry slaughterhouses in Thailand (Chotinun et al., 2014).

The challenges identified to affect laying birds in both systems provide an understanding of differences in problems faced by chicken farmers. For example, the commercial layer farmers reported lack of interaction with government officers, and the non-use of veterinarians within informal settlements making these chicken farms vulnerable to disease outbreaks. Similar disconnection with extension services have also been reported for comparable chicken systems in Uganda (FAO, 2009). The findings are useful for policy makers on disease control and management; however future research should examine the role of gender in chicken production activities which was not considered in the present study. The implementation of policies which reduce vulnerabilities within the chicken systems will not only improve food safety for consumers of products from egg laying birds, but also support livelihoods of poor households within *peri* urban areas and informal settlements which depend on chicken.

## Funding

This study was supported by the UK Medical Research Council, Biotechnology and Biological Science Research Council (UK), the Economic and Social Research Council (UK), the Natural Environment Research Council (UK), through the Environmental & Social Ecology of Human Infectious Diseases Initiative (ESEI), Grant Reference: G1100783/1. This work also received support from the CGIAR Research Program on Agriculture for Nutrition and Health (A4NH), led by the International Food Policy Research Institute (IFPRI). We also acknowledge the CGIAR Fund Donors (http://www.cgiar.org/who-we-are/cgiar-fund/fund-donors-2). Funding was also obtained from the Leverhulme Centre for Integrative Research in Agriculture and Health (London, UK).

## Declaration of interest

All the authors here declare that they have no conflict of interest in this publication.
